# 2052: Empirical assessment of a theatrical performance on attitudes and behavior intentions toward research: The informed consent play

**DOI:** 10.1017/cts.2017.188

**Published:** 2018-05-10

**Authors:** Erin Rothwell, Gretchen Case, Sydney Cheek-O’Donnell, Bob Wong, Erin Johnson, Trent Matheson, Alena Wilson, Nicole R. Robinson, Jared Rawlings, Brooke Horejsi, Jeffrey R. Botkin, Carrie L. Byington

**Affiliations:** School of Medicine, The University of Utah, Salt Lake City, UT, USA

## Abstract

OBJECTIVES/SPECIFIC AIMS: Exposure to theatrical performances holds promise for addressing bioethical issues, but there has been little empirical examination of the impact of dramatic presentation on audiences’ attitudes. This study assessed the short-term impact of the play, Informed Consent, on perceptions of trust, willingness to donate biospecimens, attitudes toward harm and privacy among the general public and in faculty, medical and undergraduate students within an academic medical center in the intermountain west. METHODS/STUDY POPULATION: Surveys were administered before and after a staged reading of the play by professional actors. Pre and post survey responses were linked for each participant. Survey items included the short form Trust in Medical Researchers, and single item questions about group identity, of genetic testing in children, and willingness to donate biospecimens. In total, 3 additional questions about harm, consent, and ethical investigator behavior as represented in the play were asked in the post survey. In addition, respondents were given the option to answer open-ended questions through email. RESULTS/ANTICIPATED RESULTS: Out of the 481 who attended the play, 421 completed both the pre and post surveys, and 166 participants completed open-ended questions online ~1 week after the play. Across all participants, there were significant declines for Trust in Medical Researchers and for the survey item “is it ethical for genetic testing in children for adult onset conditions,” (*p*<0.001 for both) following the play. There was a significant increase in agreement to improve group identity protections (*p*<0.001) and no differences on willingness to donate biospecimens to research (*p*=0.777). When differences were analyzed by race of the participant, non-White participants (n=68) compared with White participants (n=344) were less willing to donate biospecimens in general (*p*<0.001). Further, non-White participants’ willingness to donate biospecimens decreased (*p*=0.049) after viewing the play while the white participants’ willingness to donate was unchanged. Qualitative data provided extensive contextual data supporting these perspectives. DISCUSSION/SIGNIFICANCE OF IMPACT: This is one of the first studies to empirically examine the impact of a theatrical performance on both attitudes and behavioral intentions toward research and clinical research participation. Some attitudes changed following the play performance, but there were no significant differences on intention to donate biospecimens for research overall. Future research can further address the value and impact of theatrical performances and other creative arts as tools to engage the public and investigators in dialogue about the ethical issues and complexities in clinical research and further evaluation of the impact of performances on attitudes about research and ethics. Creative arts may be used to motivate investigators and study participants to confront fundamental questions about research participation and trust.

